# Meditation-State Functional Connectivity (msFC): Strengthening of the Dorsal Attention Network and Beyond

**DOI:** 10.1155/2012/680407

**Published:** 2012-02-12

**Authors:** Brett Froeliger, Eric L. Garland, Rachel V. Kozink, Leslie A. Modlin, Nan-Kuei Chen, F. Joseph McClernon, Jeffrey M. Greeson, Paul Sobin

**Affiliations:** ^1^Department of Psychiatry and Behavioral Sciences, Duke University Medical Center, Durham, NC 27708, USA; ^2^Brain Imaging and Analysis Center, Duke University Medical Center, Durham, NC 27710, USA; ^3^College of Social Work, Florida State University, Tallahassee, FL 32306-2570, USA; ^4^Trinity Institute for the Addictions, Florida State University, Tallahassee, FL 32306-2570, USA; ^5^Durham Veterans Affairs Medical Center, and VISN 6 Mental Illness Research, Education, and Clinical Center, Durham, NC 27710, USA; ^6^Duke Integrative Medicine, Duke University Medical Center, Durham, NC 27705, USA; ^7^Thousand Petals Yoga, Chapel Hill, NC 27516, USA

## Abstract

Meditation practice alters intrinsic resting-state functional connectivity (rsFC) in the default mode network (DMN). However, little is known regarding the effects of meditation on other resting-state networks. The aim of current study was to investigate the effects of meditation experience and meditation-state functional connectivity (msFC) on multiple resting-state networks (RSNs). Meditation practitioners (MPs) performed two 5-minute scans, one during rest, one while meditating. A meditation naïve control group (CG) underwent one resting-state scan. Exploratory regression analyses of the relations between years of meditation practice and rsFC and msFC were conducted. During resting-state, MP as compared to CG exhibited greater rsFC within the Dorsal Attention Network (DAN). Among MP, meditation, as compared to rest, strengthened FC between the DAN and DMN and Salience network whereas it decreased FC between the DAN, dorsal medial PFC, and insula. Regression analyses revealed positive correlations between the number of years of meditation experience and msFC between DAN, thalamus, and anterior parietal sulcus, whereas negative correlations between DAN, lateral and superior parietal, and insula. These findings suggest that the practice of meditation strengthens FC within the DAN as well as strengthens the coupling between distributed networks that are involved in attention, self-referential processes, and affective response.

## 1. Introduction

For more than two millennia, mindfulness meditation has been practiced as a means of achieving psychological equanimity and self-awareness, yet it has only recently become the target of systematic research by fields such as medicine, psychology, and neuroscience for its relevance to mental and physical health. Mindfulness meditation is becoming increasingly well regarded for its therapeutic promise [1–3]. Indeed, there is mounting empirical evidence of the role of mindfulness in reducing stress and improving clinical outcomes across issues as diverse as depression [4], irritable bowel syndrome [5], chronic pain [6], and addiction [7–9].

According to recent conceptualizations, the* practice* of mindfulness meditation (which involves repeated placement of attention onto an object while alternately acknowledging and letting go of distracting thoughts and emotions) evokes the *state* of mindfulness, which, when engaged repeatedly over time, may accrue into *trait *or* dispositional *mindfulness [10, 11]. The state of mindfulness is characterized by a nonjudgmental and metacognitive monitoring of moment-by-moment cognition, emotion, perception, and sensation without fixation on thoughts of past and future [12–14]. Correspondingly, trait mindfulness is characterized as the propensity towards exhibiting such nonjudgmental awareness of one's thoughts, emotions, experiences, and actions in everyday life [15]. Higher levels of this trait are associated with enhanced affect regulation [10], attentional control [16], and autonomic recovery from emotional provocations [17]. As a trait, mindfulness is approximately normally distributed [18]. Thus, people vary in the extent to which they exhibit mindful dispositions, yet this dispositionality can be promoted by recurrent practice of mindfulness meditation. For example, individuals participating in an eight-week Mindfulness-Based Stress Reduction course evidenced increases in trait mindfulness which mediate the effects of training on clinical outcomes [19, 20].

Thus, the therapeutic effects of mindfulness meditation may result from a *state by trait interaction*, such that recurrent activation of the mindful state (and the neural networks that instantiate this state) via the practice of mindfulness meditation may leave lasting psychobiological traces that accrue into durable changes in trait mindfulness and fundamental alterations in the sense of self [11, 21]. These changes might be mediated by experience-dependent alterations in gene expression resulting in neuroplasticity [22, 23]. In support of this hypothesis, several studies using voxel-based morphometry have identified significant differences in grey matter concentration between long-term mindfulness meditation practitioners and controls [24–26]. Moreover, recent longitudinal research suggests that participating in 8 weeks of mindfulness meditation training is associated with increases in grey matter density in the left hippocampus, posterior cingulate cortex, and temporoparietal junction, brain regions that are thought to subserve emotion regulation, learning, memory, and the ability to shift one's perspective [27]. It is possible that such changes in brain structure arise from the recurrent activation of corresponding functional networks during repeated practice of mindfulness meditation.

Neuroimaging research has demonstrated differences in task-related brain function between experienced meditation practitioners and meditation naïve controls. For example, meditation practitioners exhibit greater meditation-related neural activation in brain regions involved in attentional control (e.g., prefrontal cortex), conflict resolution (e.g., dorsal anterior cingulate cortex), and emotional processing (e.g., medial/orbitofrontal cortices) [28, 29]. However, significantly less is known regarding the effects of meditation on brain function outside of the context of explicit task-related processes.

A nascent database of neuroscience research on task-independent brain function has emerged via the study of resting-state functional connectivity (rsFC). rsFC is measured by using fMRI to examine changes in synchronized low frequency oscillations in blood-oxygen-level dependence (BOLD) signal during resting states to identify functionally interconnected brain regions [30]. Multiple resting state networks have been identified [31–33] including seven major networks: default mode (DMN), dorsal attention network (DAN), executive control (ECN), salience, sensorimotor, visual, and auditory networks [34]. The Default Mode network (DMN) [35] is spatially composed of midline regions (e.g., ventral medial prefrontal cortex (VMPFC), posterior cingulate cortex (PCC), and precuneus) and is thought to reflect nongoal directed processes [36]. Specific alterations in the spatial distribution of the DMN have been found in individuals with anxiety [37], attention deficit disorder (ADD) [38] mild cognitive impairment [39], schizophrenia [40, 41] major depressive disorder (MDD) [42], and substance abuse disorders (SUDs) [43]—suggesting that the spatial distribution of the DMN may provide important markers of both optimal and dysregulated neural functioning.

Interestingly, recent research focusing on DMN connectivity has reported that meditation practitioners, as compared to controls, exhibit greater rsFC within the DMN [44, 45] and between DMN and dlPFC [44]—a region subserving cognitive control processes [46, 47]. Furthermore, recent research demonstrates that among meditation naïve subjects, participating in 8 weeks of mindfulness meditation training increases rsFC of auditory, visual, and salience brain networks while attending to auditory information [48]. Those findings may provide important neural indices of the effects of meditation training on attention and awareness.

Though the prior research suggests that mindfulness meditation alters rsFC, a number of important questions remain. First, it remains unclear whether the differences between meditation practitioners and meditation naïve controls in rsFC extend beyond the DMN to other RSNs (e.g., executive control, dorsal attention, salience networks). To examine this question, a node-based analysis designed to evaluate cross-correlations between multiple resting state networks is ideal. Secondly, if meditation experience does indeed modulate multiple RSN, how does active meditation modulate functional connectivity among those regions? This question can be specifically addressed by measuring functional connectivity in experienced meditators during a state of nonmeditation to evaluate rsFC, and again during active meditation to evaluate meditation state functional connectivity (msFC). Finally, it is unclear whether the effects of msFC are shortterm (state-related) or if they in fact are associated with the duration of meditation practice (e.g., trait-related changes mediated by neural plasticity). Addressing these questions may provide feasibility data for evaluating the effects of mindfulness meditation training on brain function among meditation-naïve subjects. Therefore, in the current study we sought to investigate the direct effect of meditation on functional connectivity by comparing a period of active meditation with passive rest in a group of experienced meditation practitioners. In order to evaluate msFC across multiple brain regions, we implemented a node-based analytical approach [49] examining cross-correlations between regions in four major resting state networks (DMN, DAN, ECN, Salience network). In order to evaluate whether the duration of meditation practice affects the magnitude of functional connectivity across brain regions, regression analyses were performed with years of meditation experience as a predictor of rsFC and msFC strength. We hypothesized that mindfulness meditation would be associated with greater functional connectivity between multiple resting state networks.

## 2. Materials and Methods

### 2.1. Participants

 Fourteen (7 meditation practitioner (MP), 7 meditation-naïve control [CG]) participants between the ages of 18 and 55 years were enrolled. MP participants reported engaging in mindfulness meditation on average; 7 days per week (0) over the course of the previous 5.7 yrs (3.8). In addition, participants in the MP group were also involved in an active and ongoing hatha yoga practice (>45 minutes a day, three-four times per week, > three years). The matched control group reported no current or past dedicated meditation or yoga practice. In addition, all participants were right-handed, and free of any psychiatric condition or any major medical condition that would make participation unsafe or uncomfortable. Additional exclusionary criteria included current alcohol or drug abuse, use of tobacco or nicotine products, and positive urine drug screen. Female participants were required to have a negative urine pregnancy test at screening and within 12 hours prior to the fMRI scan. The protocol was approved by the institutional review board at Duke University Medical Center, and all participants provided written informed consent before participating in study-related activities.

### 2.2. fMRI Protocol

BOLD fMRI data were collected from all participants during a five-minute, eyes-closed resting-state period. In addition, immediately following the resting-state scan, MP participants were also scanned during a five-minute, eyes-closed period while engaged in meditation.

### 2.3. Image Acquisition

A 3T General Electric Signa EXCITE HD scanner (Milwaukee, WI) equipped with 40 mT/m gradients was used for image acquisition. At the start of each fMRI session, a high-resolution, three-dimensional, fast-spoiled gradient-recalled echo (3D-FSPGR) anatomical sequence was collected (FOV = 25.6 cm, matrix = 2562, flip angle = 12°, 166 slices, slice thickness = 1 mm). Blood-oxygenation-level-dependent (BOLD) functional images were collected for 34 contiguous slices (4 mm thick) parallel to the horizontal plane connecting the anterior and posterior commissures. A gradient-recalled inward spiral pulse imaging sequence was used (34 slices, TR = 1500 ms, TE = 30 ms, FOV = 24 cm, matrix = 64 × 64, flip angle = 60°, slice thickness = 4 mm, resulting in 3.75 × 3.75 × 4 mm voxels).

### 2.4. Image Preprocessing

Data were initially preprocessed in FSL: slice time correction and realignment. Motion parameters were regressed out of realigned images were then normalized to the MNI template. Following normalization, white matter and cerebral spinal fluid time series were regressed out. Data were then low-passed filtered (.08 Hz) to remove slow drift artifact.

### 2.5. Functional Connectivity Analyses

The filtered data generated were further processed with an FC analysis method [49] consisting of the following procedures.

1. fMRI data were segmented into 29, 5 mm ROI spheres around coordinates derived from a previously published template mask [34]. The nodes from the Raichle mask comprised four major brain networks: the default mode network (DMN), dorsal attention network (DAN), executive control network (ECN), and salience network.
2. The time-series signals within each ROI were averaged, so that the 4D fMRI data set (*x*, *y*, *z*, time point) was reduced to a 2D data set (29 × time point number) for each fMRI run.
3. The correlation coefficients (*r* values) between time-series data from different anatomical regions were calculated, and the calculated coefficients (Fisher *z*-transformed) were stored in a 2D matrix with 841 cells (i.e., 29 × 29, with only 29 × 28/2 unique elements). Each element of this 2D matrix represents FC between two anatomical regions. This procedure was performed for all fMRI runs acquired during passive rest and meditation in MP, and rest in CG.

## 3. Results

### 3.1. Study Participants

**Participant Demographics (see Table 1)**

Among the MP group, age was not significantly correlated with years of meditation practice (*r* = −.01, *P* = .98) or yoga (*r* = .27, *P* = .55).

### 3.2. Identification of Group Differences in Functionally Connected Regions

**Figure 1:**
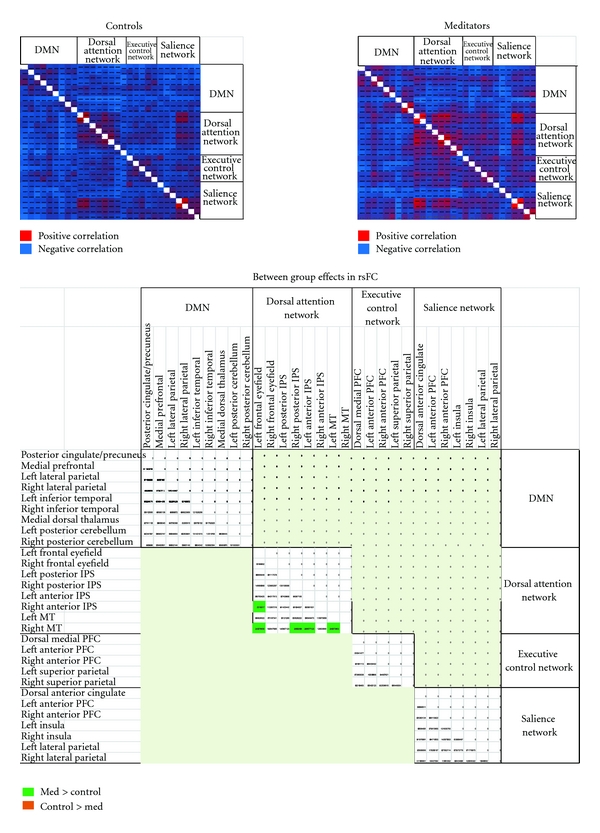
Main effects of group and group differences on rsFC.

Analyses were conducted on the interregion connectivity matrices by correlating connectivity matrix elements with a group (MP, CG) category array. In this analysis, with correlations thresholded at *t* (2-tailed) ≥ 2.18, *P* < .05, interregion connectivity matrix within nodes of the DAN (right anterior IPS and left frontal eyefield; right MT and left frontal eyefield, right posterior and left anterior IPS and left MT) was significantly higher for MP than that for CG (Figure 1). Significant findings resulted in effects sizes (Cohen's *d*) between 1.3 and 1.7.

### 3.3. The Effects of Meditation on Functionally Connected Regions

**Figure 2:**
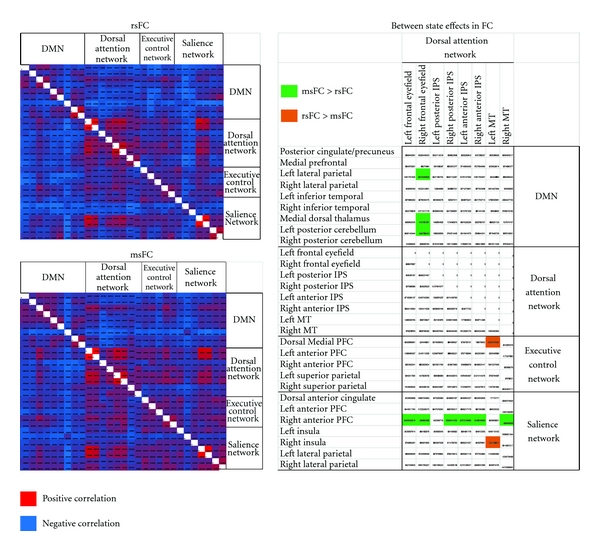
Main effects and state differences (rsFC versus msFC) in meditation practitioners.

Among MP group, analyses were conducted on the inter region connectivity matrices by correlating connectivity matrix elements of the DAN and all other networks with mindful state (meditation, resting) category array. In this analysis, with correlations thresholded at *t* (2-tailed) ≥ 2.44, *P* < .05, interregion connectivity matrix revealed meditation state functional connectivity (msFC), as compared to rsFC, to be greater between DAN (i.e., right frontal eyefield) and DMN nodes (medial dorsal thalamus, left lateral parietal and posterior cerebellum) and right anterior PFC node of the Salience Network and multiple DAN nodes (bilateral frontal eyefield, right posterior IPS, bilateral anterior IPS and right MT), whereas msFC was less than rsFC between DAN (left MT) and right insula and dorsal medial PFC (Figure 2). Significant findings resulted in effects sizes (Cohen's *d*) between 2.1 and 3.3.

### 3.4. Relations between Meditation Experience and Functional Connectivity during Resting State and Meditation

Regression analyses were conducted on the inter-region connectivity matrices by correlating connectivity matrix elements during resting state and during meditation, entering years of meditation practice as a regressor. In these analyses, correlations were thresholded at rZ.996 (*P* < .005) which corresponds to *r* = .71.

**Figure 3:**
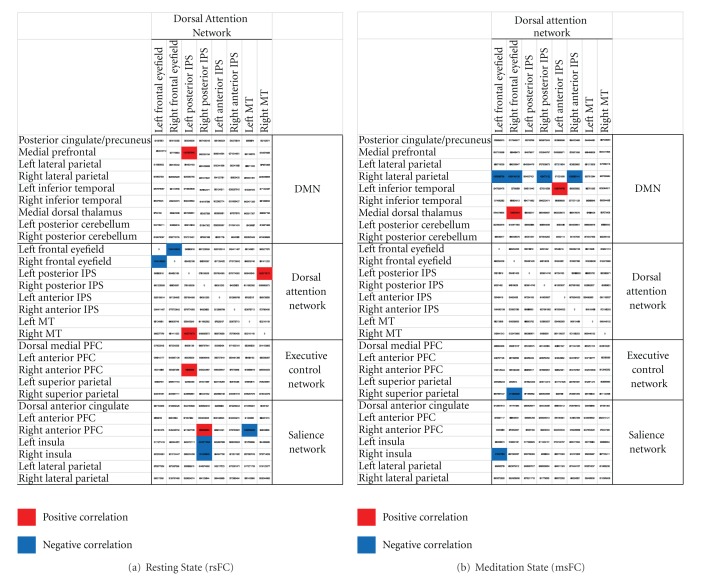
Results from meditation years regressed against (a) resting state and (b) meditation state functional connectivity.

During rest, meditation experience predicted strengthening of rsFC within the DAN (between left posterior IPS and right MT) and between DAN (i.e., left posterior IPS) and DMN (i.e., medial PFC), ECN (i.e., right anterior PFC) and salience network (i.e., right anterior PFC), but weakening between right posterior IPS and bilateral insula, and left MT and right anterior PFC (Figure 3(a)).

With regard to msFC, meditation experience predicted strengthening of msFC between DAN and DMN (i.e., right frontal eyefield with medial dorsal thalamus; left anterior IPS with left inferior temporal), but a pattern of weakening between right lateral parietal node of the DMN and 50% of the nodes in the DAN (bilateral frontal eyefiel, right posterior, and anterior IPS), as well as left eyefield and right insula, right eyefield, and right superior parietal (Figure 3(b)). 

## 4. Discussion

Neuroimaging results supported both study hypotheses: mindfulness meditation practitioners (MPs) exhibited significantly greater rsFC than meditation naïve individuals in the control group (CG) in the dorsal attention network (DAN), and mindfulness meditation practice in the scanner was associated with increased functional connectivity from resting state levels (i.e., msFC > rsFC) between the DAN and DMN and right PFC node of the Salience network. Together, these findings from the present study suggest that mindfulness practice enhances functional connectivity within attentional networks as well as increases connectivity across broadly distributed brain regions subserving attention, self-referential, and emotional processes. The findings from the present study demonstrate feasibility for measuring task-independent neural function associated with meditation. Moreover, they provide a methodological approach that may be utilized to evaluate the effects of mindfulness meditation training in a larger sample of meditation-naïve subjects.

### 4.1. MP versus CG Differences

Our observation of elevated levels of rsFC among MP compared with non-meditators extends the previous research on the effects of meditation on DMN connectivity [44, 45] by demonstrating that MP exhibit strengthened rsFC in the DAN. It is possible that these increases in rsFC reflect increased trait mindfulness and attentional control stemming from long-term meditation practice. Indeed, compared with nonmeditators, MPs evidence greater rsFC between the dorsal attention network, DMN, and salience network, which may index allocation of attentional resources toward enhanced self-reflection and awareness of emotional experience. Alternatively, such differences in rsFC may be correlated with traits associated with the motivation to engage in long-term meditation practice.

### 4.2. Active Meditation Alters Functional Connectivity

Among MPs, we observed increased FC during active meditation, as opposed to rest. Notably, stronger functional coupling was observed between the DAN, DMN, and salience network. Such increased functional connectivity between DAN and DMN may be meaningfully contrasted with decreased DMN rsFC often observed in populations with cognitive or emotional disturbances including attention deficit disorder (ADD) [38], mild cognitive impairment [39], schizophrenia [40, 41], major depressive disorder (MDD) [42], and substance abuse disorders (SUDs) [43]. In this sense, mindfulness meditation appears to involve a shift from a functionally restricted default mode during rest into a more functionally integrated large-scale network subserving attention, salience, and self-reflection.

 This state-related shift in FC may reflect the practice of mindfulness meditation, which first involves becoming aware of when attention has wandered from the object of meditation (often a sensorimotor input such as the sensation of breathing) into self-relevant or emotionally salient thoughts, feelings, images, and memories [14]. These mental contents are then apprehended with an attitude of acceptance and nonjudgment. Subsequently, the meditator disengages his or her attentional focus and reorients attention back to the meditative object. Thus, the complex act of meditation may involve attentional reorienting, self-monitoring, and emotion regulation. As such, the practice of mindfulness meditation may require functional integration of multiple brain networks to coordinate these subcomponent processes into a coherent mental operation. Furthermore, functional coupling of the DAN, DMN, and anterior PFC, which has been implicated in metacognition [50] and mind-wandering (Christoff, in press), might subserve the attentional regulation and open monitoring of thoughts and emotions held to be central to mindfulness meditation [14].

### 4.3. Meditation Experience Increases the Magnitude of FC

Regression analyses identified a statistically significant association between number of years of meditation experience and rsFC between the DAN and the four major networks examined (i.e., DAN, DMN, ECN, and salience). Namely, a number of positive correlations were observed between DAN and medial and dorsolateral prefontal regions, whereas negative correlations were observed between DAN and bilateral insula.

In addition, meditation experience predicted strengthening of msFC between DAN, thalamus, and inferior temporal nodes of the DMN. Inferior temporal lobe may be the node of the ventral allocentric stream of visuospatial reference, that is, responsible for object processing in relation to the environment [51]. Allocentric processing is in contradistinction to the more dorsal egocentric stream, which performs object processing in relation to the self [52]. The finding that FC in these networks increased with meditation experience may correspond with accounts of long-term mindfulness practitioners who report experiencing thoughts and feelings as objects without any self-reference [21]. With the deepening of meditative practice over time, this experience is held to result in the sense of self as emptiness (sunyata) and the nonduality of “the inner space of the mind and the outer space of objective phenomena” [53, page 219].

Both at rest and during active meditation, long-term practitioners of mindfulness meditation evidenced greater FC between the DAN and other brain networks than their less experienced counterparts. The association between meditation experience and FC parallels the greater rsFC observed among meditators as compared to nonmeditators. These findings imply the presence of a state by trait interaction, such that brain regions which are functionally coupled during the state of mindfulness become more tightly coupled over time, even during the nonmeditative state. Purportedly, long-term practitioners achieve progressively deeper altered states of consciousness during meditation that come to influence daily life experience outside of meditation [21]. Such changes in FC may be reflective of increased trait mindfulness, cortical and subcortical remodeling via neuroplasticity, and fundamental changes to the sense of self over time resulting from repeated mindfulness practice.

## 5. Conclusions and Limitations

 The present study included a well-controlled matched sample of meditation practitioners and meditation naive subjects, a neuroimaging paradigm that allows for modeling direct effects of meditation on brain function outside of the context of task-dependent neural processes—areas of research currently underrepresented in the literature. This is the first study to our knowledge to identify the effects of meditation on multiple resting state networks—providing both novel findings and feasibility for subsequent investigation in a larger sample. However, limitations include a relatively small sample size, liberal significance threshold, limited characterization of individual differences (e.g., personality), relatively short duration of meditation in the scanner, and the fact that meditation practitioners were scanned twice, whereas controls were only scanned once. Subsequent work in our laboratory will seek to evaluate the effects of meditation on state and trait mindfulness and explore relations with brain structure and function. For example, future research might profit from examining correlations between rsFC and changes in grey matter volume. We hypothesize that enhanced rsFC among long-term mindfulness practitioners would be significantly associated with increased grey matter volume in the functionally integrated neural structures of the networks identified by the present study. Furthermore, we would expect that such increases in rsFC and grey matter density would be correlated with changes in trait mindfulness and altered performance on tasks involving attention, self-monitoring, and emotion regulation. To test these hypotheses, longitudinal studies are needed to follow individuals as they initiate the discipline of mindfulness meditation and gradually develop expertise over months or years of recurrent practice. 

##  Funding

F. Joseph McClernon is funded by the NIDA Grant DA023516.

## Figures and Tables

**Table 1:** Participant demographics.

	Meditators (*N* = 7)	Controls (*N* = 7)
% Female	86%	86%
Mean Age (SD)	36.4 (11.9)	35.5 (7.1)
Years of Education (SD)	15.5 (2.5)	15.3 (2.3)
Years of Yoga (SD)	9.3 (2.4)	0
Years of Meditation (SD)	5.6 (4.2)	0

## References

[1] Chiesa A, Serretti A (2009). Mindfulness-based stress reduction for stress management in healthy people: a review and meta-analysis. *Journal of Alternative and Complementary Medicine*.

[2] Greeson JM (2009). Mindfulness research update 2008. *Complementary Health Practice Review*.

[3] Ludwig DS, Kabat-Zinn J (2008). Mindfulness in medicine. *Journal of the American Medical Association*.

[4] Teasdale JD, Moore RG, Hayhurst H, Pope M, Williams S, Segal ZV (2002). Metacognitive awareness and prevention of relapse in depression: empirical evidence. *Journal of Consulting and Clinical Psychology*.

[5] Gaylord SA, Palsson OS, Garland EL (2011). Mindfulness training reduces the severity of irritable bowel syndrome in women: results of a randomized controlled trial. *American Journal of Gastroenterology*.

[6] Rosenzweig S, Greeson JM, Reibel DK, Green JS, Jasser SA, Beasley D (2010). Mindfulness-based stress reduction for chronic pain conditions: variation in treatment outcomes and role of home meditation practice. *Journal of Psychosomatic Research*.

[7] Bowen S, Chawla N, Collins SE (2009). Mindfulness-based relapse prevention for substance use disorders: a pilot efficacy trial. *Substance Abuse*.

[8] Bowen S, Marlatt A (2009). Surfing the urge: brief mindfulness-based intervention for college student smokers. *Psychology of Addictive Behaviors*.

[9] Garland EL, Gaylord SA, Boettiger CA, Howard MO (2010). Mindfulness training modifies cognitive, affective, and physiological mechanisms implicated in alcohol dependence: results of a randomized controlled pilot trial. *Journal of Psychoactive Drugs*.

[10] Chambers R, Gullone E, Allen NB (2009). Mindful emotion regulation: an integrative review. *Clinical Psychology Review*.

[11] Garland EL, Fredrickson B, Kring AM, Johnson DP, Meyer PS, Penn DL (2010). Upward spirals of positive emotions counter downward spirals of negativity: Insights from the broaden-and-build theory and affective neuroscience on the treatment of emotion dysfunctions and deficits in psychopathology. *Clinical Psychology Review*.

[12] Garland EL (2007). The meaning of mindfulness: a second-order cybernetics of stress, metacognition, and coping. *Complementary Health Practice Review*.

[13] Kabat-Zinn J (1982). An outpatient program in behavioral medicine for chronic pain patients based on the practice of mindfulness meditation: theoretical considerations and preliminary results. *General Hospital Psychiatry*.

[14] Lutz A, Slagter HA, Dunne JD, Davidson RJ (2008). Attention regulation and monitoring in meditation. *Trends in Cognitive Sciences*.

[15] Baer RA, Smith GT, Hopkins J, Krietemeyer J, Toney L (2006). Using self-report assessment methods to explore facets of mindfulness. *Assessment*.

[16] Moore A, Malinowski P (2009). Meditation, mindfulness and cognitive flexibility. *Consciousness and Cognition*.

[17] Garland EL (2011). Trait mindfulness predicts attentional and autonomic regulation of alcohol cue-reactivity. *Journal of Psychophysiology*.

[18] Walach H, Buchheld N, Buttenmüller V, Kleinknecht N, Schmidt S (2006). Measuring mindfulness-the Freiburg Mindfulness Inventory (FMI). *Personality and Individual Differences*.

[19] Carmody J, Baer RA (2008). Relationships between mindfulness practice and levels of mindfulness, medical and psychological symptoms and well-being in a mindfulness-based stress reduction program. *Journal of Behavioral Medicine*.

[20] Greeson JM, Webber DM, Smoski MJ (2011). Changes in spirituality partly explain health-related quality of life outcomes after Mindfulness-Based Stress Reduction. *Journal of Behavioral Medicine*.

[21] Austin JH (2009). *Zen and the Meditative Transformations of Consciousness*.

[22] Garland EL, Howard MO (2009). Neuroplasticity, psychosocial genomics, and the biopsychosocial paradigm in the 21st century. *Health and Social Work*.

[23] Dusek JA, Otu HH, Wohlhueter AL (2008). Genomic counter-stress changes induced by the relaxation response. *PLoS One*.

[24] Hölzel BK, Ott U, Gard T (2008). Investigation of mindfulness meditation practitioners with voxel-based morphometry. *Social Cognitive and Affective Neuroscience*.

[25] Lazar SW, Kerr CE, Wasserman RH (2005). Meditation experience is associated with increased cortical thickness. *NeuroReport*.

[26] Luders E, Toga AW, Lepore N, Gaser C (2009). The underlying anatomical correlates of long-term meditation: larger hippocampal and frontal volumes of gray matter. *NeuroImage*.

[27] Hölzel BK, Carmody J, Vangel M (2011). Mindfulness practice leads to increases in regional brain gray matter density. *Psychiatry Research*.

[28] Short EB, Kose S, Mu Q (2010). Regional brain activation during meditation shows time and practice effects: an exploratory FMRI study. *Evidence-based Complementary and Alternative Medicine*.

[29] Hölzel BK, Ott U, Hempel H (2007). Differential engagement of anterior cingulate and adjacent medial frontal cortex in adept meditators and non-meditators. *Neuroscience Letters*.

[30] Beckmann CF, DeLuca M, Devlin JT, Smith SM (2005). Investigations into resting-state connectivity using independent component analysis. *Philosophical Transactions of the Royal Society B*.

[31] Biswal BB, Van Kylen J, Hyde JS (1997). Simultaneous assessment of flow and BOLD signals in resting-state functional connectivity maps. *NMR in Biomedicine*.

[32] Seeley WW, Menon V, Schatzberg AF (2007). Dissociable intrinsic connectivity networks for salience processing and executive control. *Journal of Neuroscience*.

[33] Tomasi D, Volkow ND (2011). Functional connectivity hubs in the human brain. *NeuroImage*.

[34] Raichle ME (2011). The restless brain. *Brain Connectivity*.

[35] Raichle ME, MacLeod AM, Snyder AZ, Powers WJ, Gusnard DA, Shulman GL (2001). A default mode of brain function. *Proceedings of the National Academy of Sciences of the United States of America*.

[36] Raichle ME, Snyder AZ (2007). A default mode of brain function: a brief history of an evolving idea. *NeuroImage*.

[37] Zhao XH, Wang PJ, Li CB (2007). Altered default mode network activity in patient with anxiety disorders: an fMRI study. *European Journal of Radiology*.

[38] Castellanos FX, Margulies DS, Kelly C (2008). Cingulate-precuneus interactions: a new locus of dysfunction in adult attention-deficit/hyperactivity disorder. *Biological Psychiatry*.

[39] Sorg C, Riedl V, Mühlau M (2007). Selective changes of resting-state networks in individuals at risk for Alzheimer’s disease. *Proceedings of the National Academy of Sciences of the United States of America*.

[40] Liang M, Zhou Y, Jiang T (2006). Widespread functional disconnectivity in schizophrenia with resting-state functional magnetic resonance imaging. *NeuroReport*.

[41] Liu Y, Liang M, Zhou Y (2008). Disrupted small-world networks in schizophrenia. *Brain*.

[42] Veer IM, Beckmann CF, van Tol M (2010). Whole brain resting-state analysis reveals decreased functional connectivity in major depression. *Frontiers in Systems Neuroscience*.

[43] Ma N, Liu Y, Li N (2010). Addiction related alteration in resting-state brain connectivity. *NeuroImage*.

[44] Brewer JA, Worhunsky PD, Gray JR, Tang Y-Y, Weber J, Kober H (2011). Meditation experience is associated with differences in default mode network activity and connectivity. *Proceedings of the National Academy of Sciences of the United States of America*.

[45] Jang JH, Jung WH, Kang DH (2011). Increased default mode network connectivity associated with meditation. *Neuroscience Letters*.

[46] Cabeza R, Nyberg L (2000). Imaging cognition II: an empirical review of 275 PET and fMRI studies. *Journal of Cognitive Neuroscience*.

[47] Egner T, Delano M, Hirsch J (2007). Separate conflict-specific cognitive control mechanisms in the human brain. *NeuroImage*.

[48] Kilpatrick LA, Suyenobu BY, Smith SR (2011). Impact of mindfulness-based stress reduction training on intrinsic brain connectivity. *NeuroImage*.

[49] Chen NK, Chou YH, Song AW, Madden DJ (2009). Measurement of spontaneous signal fluctuations in fMRI: adult age differences in intrinsic functional connectivity. *Brain structure &amp; function*.

[50] Stuss DT (2011). Functions of the frontal lobes: relation to executive functions. *Journal of the International Neuropsychological Society*.

[51] Medina J, Kannan V, Pawlak MA (2009). Neural substrates of visuospatial processing in distinct reference frames: evidence from unilateral spatial neglect. *Journal of Cognitive Neuroscience*.

[52] Volcic R, Kappers AML (2008). Allocentric and egocentric reference frames in the processing of three-dimensional haptic space. *Experimental Brain Research*.

[53] Wallace BA, Hodel B (2008). *Embracing Mind: The Common Group of Science & Spirituality*.

